# Cloning, expression and biochemical characterization of the cholesterol oxidase CgChoA from *Chryseobacterium gleum*

**DOI:** 10.1186/1472-6750-14-46

**Published:** 2014-05-21

**Authors:** Renate Reiss, Greta Faccio, Linda Thöny-Meyer, Michael Richter

**Affiliations:** 1 Empa. Swiss Federal Laboratories for Materials Science and Technology, Laboratory for Biomaterials, Lerchenfeldstr. 5, 9014 St. Gallen, Switzerland

**Keywords:** *Chryseobacterium gleum*, Cholesterol oxidase, Recombinant expression in *Escherichia coli*, Biocatalysis, Taurocholate

## Abstract

**Background:**

Cholesterol oxidases are important enzymes for applications such as the analysis of cholesterol in clinical samples, the synthesis of steroid derived drugs, and are considered as potential antibacterial drug targets.

**Results:**

The gene *choA* encoding a cholesterol oxidase from *Chryseobacterium gleum* DSM 16776 was cloned into the pQE-30 expression vector and heterologously expressed in *Escherichia coli* JM109 co-transformed with pRARE2. The N-terminally His-tagged cholesterol oxidase (CgChoA) was assigned to be a monomer in solution by size exclusion chromatography, showed a temperature optimum of 35°C, and a pH optimum at 6.75 using 0.011 M MOPS buffer under the tested conditions. The purified protein showed a maximum activity of 15.5 U/mg. CgChoA showed a Michaelis-Menten like kinetic behavior only when the substrate was dissolved in water and taurocholate (apparent *K*_m_ = 0.5 mM). In addition, the conversion of cholesterol by CgChoA was studied *via* biocatalytic batches at analytical scale, and cholest-4-en-3-one was confirmed as product by HPLC-MS.

**Conclusion:**

CgChoA is a true cholesterol oxidase which activity ranges among the high performing described cholesterol oxidases from other organisms. Thus, the enzyme broadens the available toolbox of cholesterol oxidases for e.g. synthetic and biosensing applications.

## Background

**Figure 1:**
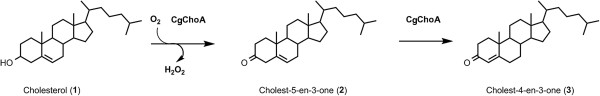
Reaction scheme for the oxidation of cholesterol catalyzed by CgChoA.

Cholesterol oxidase (EC 1.1.3.6, ChoA) is a FAD-dependent bifunctional enzyme that catalyzes the oxidation and isomerization of cholesterol (Figure 1, **1**) to cholest-4-en-3-one (**3**) while dioxygen is finally reduced to H_2_O_2_ as by-product. The enzymatic overall cholesterol oxidation comprises three steps. In the first one the 3β-OH group of cholesterol is oxidized to the corresponding ketone (**2**) with the concomitant reduction of the FAD cofactor. In a second step an isomerization of the double bond from the Δ5-6 position to the Δ4-5 position takes place. The FAD is recycled in a redox reaction with dioxygen, yielding hydrogen peroxide (Figure 1) [1,2]. The substrate range of described ChoA enzymes is not exclusively bound to cholesterol and the conversion of methanol, propan-2-ol and allylic alcohols has been described [3,4]. The overall enzyme structure comprises two domains, the FAD binding domain and the substrate binding domain. FAD can either be bound non-covalently (Class I) or linked covalently to a histidine residue of the apoprotein (Class II). A conserved FAD-binding sequence (GxGxGxxxxAxxxxxxG) has been described in the N-terminal region of ChoA from *Streptomyces* sp., *Brevibacterium* sp*.*, and *Rhodococcus equi*[5]. However, the overall amino acid sequences of the two classes do not show high homology [6]. Cholesterol oxidases are found exclusively in bacteria and have been described in various species including *Brevibacterium* sp., *Nocardia erythropolis, Streptomyces* sp., *Rhodococcus* sp*.*, and *Pseudomonas fluorescens.* The enzymes from these organisms are all commercially available. In some cases the enzyme is secreted (*Streptomyces* spp., *Chromobacterium* sp.), but it can also be membrane-bound (*Rhodococcus rhodochrous*), or be produced intracellularly (*Mycobacterium* spp.). The enzyme from *Brevibacterium* sp. has been expressed recombinantly in *E. coli* and in *Streptomyces lividans*[3,7]. Cholesterol oxidase producers can be divided into non-pathogenic bacteria, which use cholesterol as carbon and energy source, and pathogenic bacteria, which utilize cholesterol oxidase for infection by converting the cholesterol of membranes, thus causing damage by altering the physical structure of the membrane [8]. Therefore, and since no eukaryotic enzyme homologues exist, this type of bacterial cholesterol oxidase qualifies as potential target for a new class of antibiotics [9]. An important diagnostic application of the enzyme is the determination of cholesterol in food and blood serum with electrochemical biosensors and based on a cholesterol oxidase-peroxidase coupled reaction [10,11]. The enzyme has also been found to be insecticidal, and transgenic plants have been designed with *in situ* insecticide activity [12]. Moreover, the enzyme has been used as biocatalyst in the synthesis of high value intermediates for industrial steroid drug production and also as tool for studying biological membranes [8,13].

It has been reported that in biotransformation reactions whole cells of *Chryseobacterium gleum* were successfully used for the biotransformation of cholesterol to androsta-1,4-diene-3,17-dione, which is a precursor of antifertility drugs (e.g. estrogens), androgens and the diuretic drug spironolactone [14,15]. This strain might therefore be an ideal candidate for strain engineering in order to optimize such biotransformation approaches. In this study, a novel cholesterol oxidase from *Chryseobacterium gleum* DSM 16776 (CgChoA) was cloned, expressed in *E. coli*, purified, and biochemically characterized. Moreover, we confirm that enzymatic reactions with purified CgChoA and cholesterol as substrate yields cholest-4-en-3-one as reaction product by HPLC-MS analysis. The isolated enzyme might thus be useful in fields focused on the biosensing of cholesterol.

## Results

### *In silico* amino acid analysis of ChoA variants

For the identification of a novel bacterial cholesterol oxidase, a Protein Blast search was performed using the cholesterol oxidase amino acid sequence from *Streptomyces* sp. (UniProt accession number P12676; PDB code 2GEW) as template. Protein sequences of ChoA were retrieved from public databases, aligned using the ClustalW algorithm of the MegAlign software (LASERGENE, Madison, USA), and analyzed in order to identify conserved residues possibly important for the catalytic activity. Out of numerous homologues, the gene *choA* encoding a hypothetical protein (CgChoA) annotated as cholesterol oxidase was found in the fully sequenced genome of *Chryseobacterium gleum* ATCC 35910 (DSM 16776; UniProt accession number D7VYA1). The gene was selected for cloning and recombinant expression in *E. coli*.

**Figure 2:**
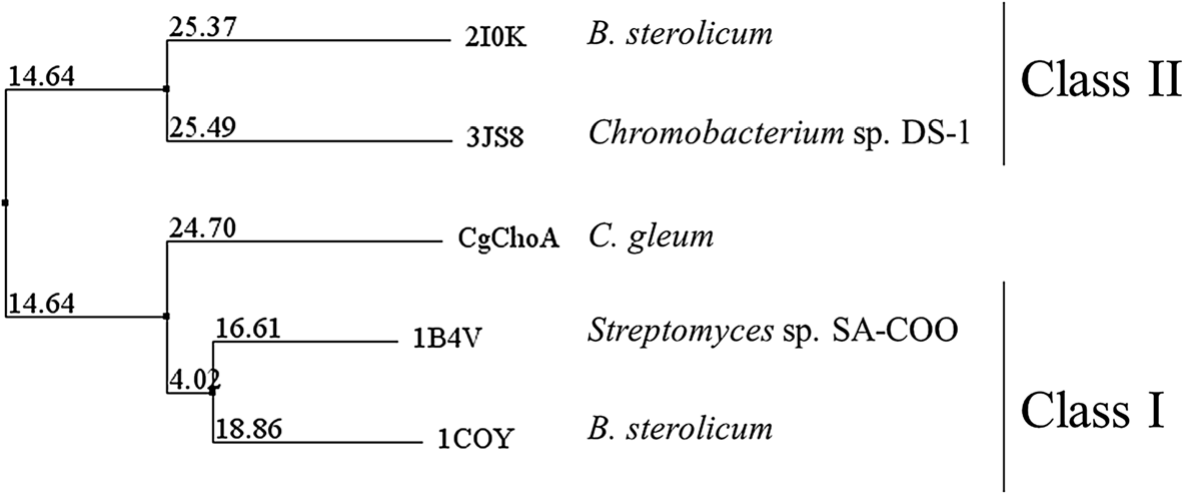
**Phylogenetic tree of CgChoA and selected characterized cholesterol oxidases.** The unrooted tree was based on the average distance PID and reports the percentage of identity between each protein sequence *vs*. the consensus sequence generated from the alignment of all the protein sequences considered. In addition to the enzyme CgChoA (subject of this study), the cholesterol oxidases considered are indicated by their PDB identifier and are the (1COY, cofactor non-covalently bound) and the (2I0K, cofactor covalently bound) flavoproteins from *Brevibacterium sterolicum*, the flavoprotein from *Streptomyces* sp. SA-COO (1B4V, cofactor non-covalently bound), and the flavoprotein from *Chromobacterium* sp. DS-1 (3JS8 cofactor covalently bound). The tree was produced with the software Jalview ( http://www.jalview.org).

The amino acid sequence of CgChoA carries the typical sequence of the Rossmann fold (xh)_2_GxGxxGx(xxh)_2_(x)_8_hxhE, where x is any amino acid and h an hydrophobic one, between V44 and E70 in the N-terminal region. This indicated that CgChoA is an FAD-binding protein [2]. Alignment to selected well-studied cholesterol oxidases and phylogenetic analysis indicated a higher similarity of CgChoA to the non-covalent FAD-dependent enzymes belonging to the Class I family (Figure 2). The lack of a signal peptide indicated the intracellular localization of the enzyme in the native host. Using sequence alignment, CgChoA was analyzed for the presence of residues reported to be important for the catalytic activity [4]. More in detail, residues N485 and Y446 (N522 and Y483 in the original protein sequence GenBank: AAA26719.1, respectively) reported to contribute to the stabilization of the cofactor in the reduced form in the cholesterol oxidase from *Streptomyces* sp. SA-COO were found conserved in CgChoA, e.g. N503 and Y464. Similarly, amino acid E398, corresponding to E361 (E402) in the cholesterol oxidase from *Streptomyces* sp. SA-COO, that is supposedly involved in the catalytic process by facilitating deprotonation of the substrate was conserved in CgChoA.

The cDNA sequence encoding CgChoA was cloned into the expression vector pQE-30 such that the final construct pCgChoA coded for an N-terminal His-tag MRGSHHHHHHGSAC fused to CgChoA. The wild-type CgChoA amino acid sequence (without the His-tag) of *C. gleum* DSM 16776 (UniProt D7VYA1, 528 aa) showed 46.1% identity to that from *Streptomyces* sp*.* (PDB code 2GEW, 540 aa), 42.8% identity to that from *B. sterolicum* (UniProt P22637), 16.1% to that from *Mycobacterium tuberculosis* (PDB code 2XKR, 398 aa) and 14.1% to that from *Chromobacterium* sp*.* (PDB code 3JS8, 587 aa). The CgChoA cholesterol oxidase with the N-terminal His-tag consists of 541 amino acids and has a hypothetical molecular mass of 60.4 kDa.

### Expression of cholesterol oxidase from *C. gleum choA* in *E. coli*

The gene *choA* from *C. gleum* DSM 16776 contains 8% rare codons with respect to the codon usage of *E. coli*. Therefore, the expression host *E. coli* JM109 was additionally transformed with the pRARE2 plasmid, which encodes extra copies of genes coding for tRNAs recognizing the codons AGG, AGA, AUA, CUA, CCC, GGA and CGG. *E. coli* JM109 cells producing CgChoA in the absence of pRARE2 showed only low activity. In the presence of pRARE2, the *choA* gene was expressed at 30°C, but the protein was found in inclusion bodies. Activity could only be detected in the insoluble fractions. Only when the cultivation temperature was decreased to 16°C immediately after induction, soluble and active protein was present.

### Protein purification and characterization

**Figure 3:**
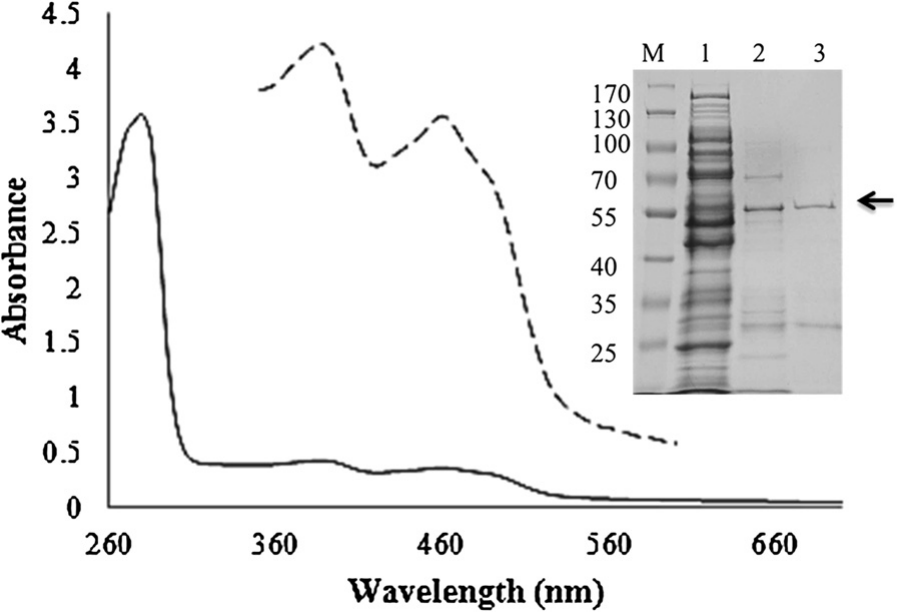
**UV–vis absorbance spectrum of purified CgChoA.** The typical absorption spectra for FAD was obtained (peaks at 370 nm and 470 nm; dashed line: 10 fold magnification) [17]. Inset: SDS-PAGE before and after purification of CgChoA. Lane 1: cell free extract; lane 2: protein after IMAC purification; lane 3: purified sample after SEC. The band corresponding to CgChoA is indicated by the arrow.

Protein purification was carried out using a Ni-affinity chromatography and subsequently a size exclusion chromatography step. The apparent molecular mass of the expressed CgChoA was around 60 kDa, when visualized on a SDS-polyacrylamide gel (Figure 3). Yields of around 0.2 mg/L culture of purified and enriched CgChoA were usually obtained. Protein bands obtained in SDS-PAGE were analyzed by tryptic digestion, subsequent MS analyses, and *in silico* processing using Mascot search program (Functional Genomics Center Zürich). The band above 55 kDa was confirmed to be CgChoA by tryptic digest and MALDI-MS/MS. The lower band of approximately 30 kDa of lane 3 (Figure 3) was found to be a houskeeping transferase and isomerase as proven by tryptic digestion and MALDI MS/ESI-MS. The molecular mass of the native protein CgChoA in solution was estimated to be about 85 kDa by size exclusion chromatography on a Superdex 200 pg column. The estimated mass was somewhat higher than 60 kDa, but lower than for a theoretical dimer with 120 kDa, which indicates that the functional enzyme is rather a monomer than a dimer in solution.

**Figure 4:**
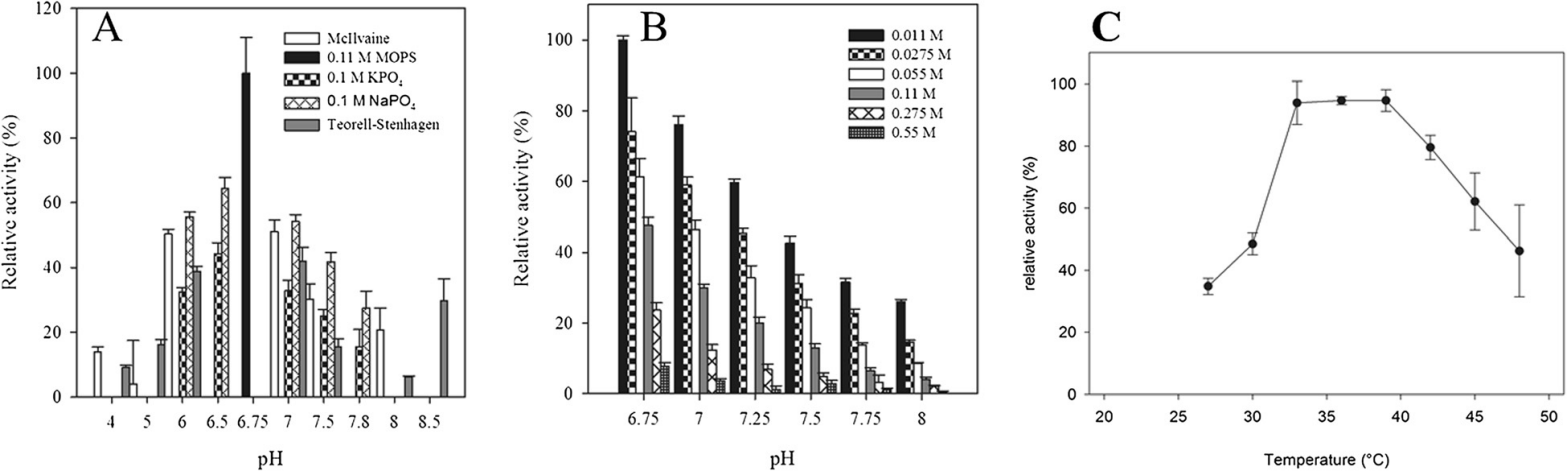
**Data obtained from a pH and buffer screen using partially purified cholesterol oxidase from *C. gleum*. (A)** pH and buffer screen using partially purified cholesterol oxidase from *C. gleum* at 25°C. The effect of pH and buffer composition upon initial rates of cholesterol oxidation using the HRP coupled cholesterol oxidation assay with ABTS as electron donor was investigated. All data points represent mean values ± SEM from triplicate determinations. 100% corresponds to 3.8 U/L. **(A)** pH and buffer screen at 25°C. **(B)** MOPS buffer screen using partially purified cholesterol oxidase from *C. gleum* at 25°C The effect of pH and molarity upon initial rates of cholesterol oxidation using the HRP coupled cholesterol oxidation assay with ABTS as electron donor was investigated. All data points represent mean values ± SEM from triplicate determinations. 100% corresponds to 29.6 U/L. **(C)** Temperature dependency study of cholesterol oxidase activity from *C. gleum* in 0.11 M MOPS buffer, pH 6.75. The oxidation of cholesterol as a function of temperature is given relative to the highest activity recorded (47.0 U/L) that was taken as 100%. All data points represent mean values ± SEM from triplicate determinations.

Purified CgChoA had a yellow colour and its spectrum showed the characteristic absorbance peaks of flavin-binding proteins (Figure 3). Heat-treatment was used to assess the possible covalent binding of the flavin cofactor to CgChoA apoprotein [16]. The purified enzyme sample after size exclusion chromatography was boiled in the dark for 5 min and centrifuged. A spectrum of the supernatant was recorded between 260–700 nm and showed a typical pattern of an FAD spectrum with two absorption maxima at 370 nm and 470 nm [17]. Only FAD that is non-covalently associated with the enzyme is detectable by this method, as covalently bound FAD co-precipitates with the protein [16].After Ni-affinity chromatography, the partially purified protein was subjected to a pH-screen for best activity in different buffers. First, various buffers were tested as shown in Figure 4 (A). As the enzyme performed using 0.11 M MOPS buffer, this buffer was tested between pH 6–10 and at molarities between 0.55 M and 0.011 M. It was found that cholesterol oxidase activity in the coupled assay was highest using 0.011 M MOPS at pH 6.75, as shown in Figure 4 (B). All subsequent measurements were therefore performed in this buffer. A temperature dependency study was also performed in a similar way. CgChoA maximum activity was measured at around 35°C (Figure 4C). The pH, molarity and temperature screens were performed with cholesterol oxidase from different purification batches that had been stored for different periods prior to use. Calculated volumetric activities as presented in Figure 4 can therefore not be compared directly. However, the overall trend is valid.

**Figure 5:**
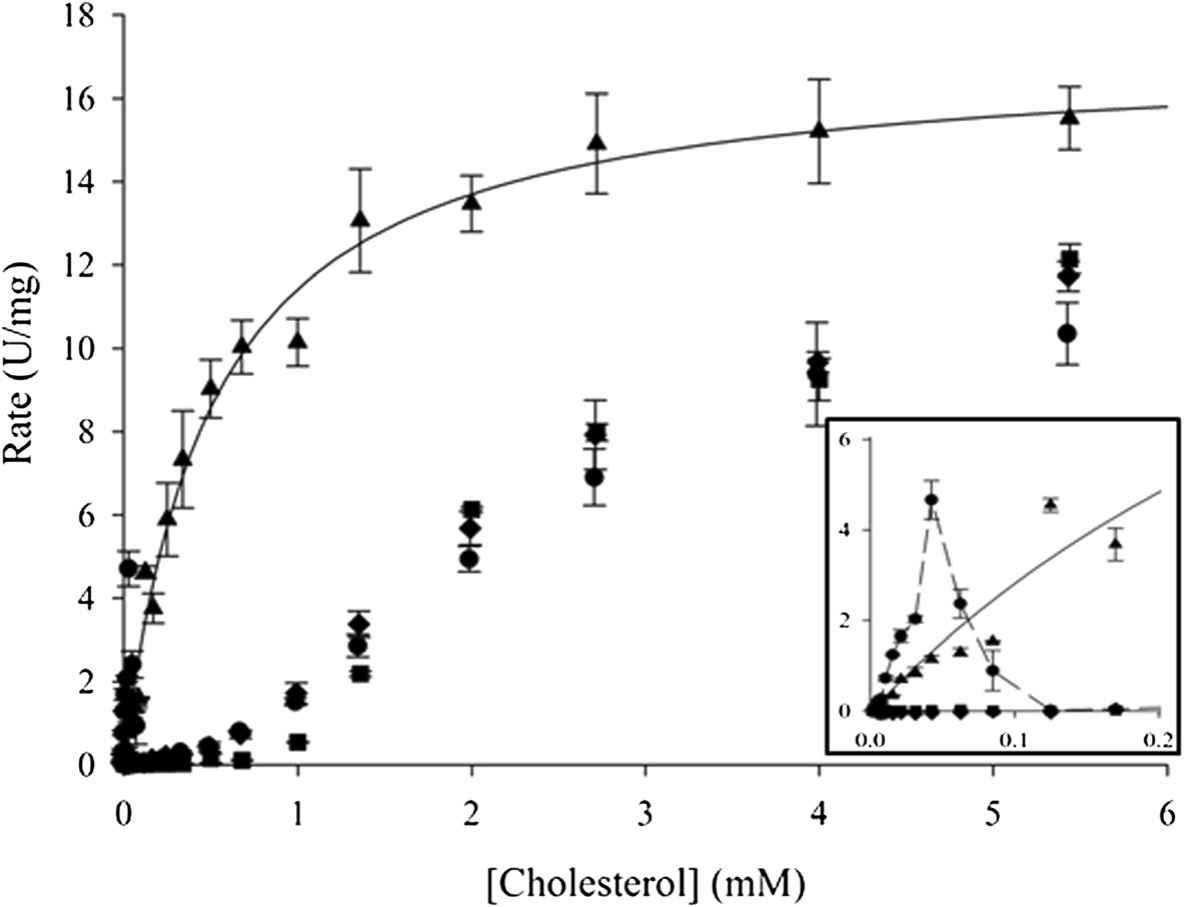
**Kinetic plot obtained with recombinant CgChoA from *C. gleum.*** The specific activity (U/mg) was plotted *versus* the substrate concentration. An enzyme concentration of 3.57 mg/L was used and the cholesterol stock solution/dispersion was prepared and serially diluted in (●) water only, (▲) water and taurocholate, (♦) water containing 5% Triton X-100 and taurocholate, or in (■) water containing 5% (v/v) Triton X-100.

The cholesterol-oxidizing activity of purified CgChoA was assayed at 35°C using 0.011 M MOPS, pH 6.75 buffer in a horseradish peroxidase (HRP) coupled assay. 23 cholesterol solutions from 0.17 μM to 5.5 mM were prepared and CgChoA initial activity was determined. We tested ABTS, pyrogallol red and *o*-dianisidine as hydrogen peroxidase substrates and found only minor changes. However, the amount of co-solvent had a significant influence. As control also *E. coli* JM109 cells transformed with the pQE-30 vector as empty vector control were tested and additionally the *E. coli* JM109 transformed with pCgChoA after incubation and induction with IPTG as described. After lysis of the cells no conversion of cholesterol could be detected in the empty vector control. No Michaelis-Menten behaviour was found for CgChoA preparations using cholesterol prepared and diluted in: only water, water with Triton X-100, and water with Triton X-100 and taurocholate, and in these cases and sigmoidal-like curve was obtained when plotting the data obtained (Figure 5). When the substrate was prepared and diluted in water and taurocholate as sole surfactant, a Michaelis-Menten like curve could be fitted and an apparent kinetic constant *K*_m_ of 0.5 mM was obtained. For the cholesterol dispersions diluted in water only, a bell-shape profile of the data between 0–0.125 mM cholesterol (see also inset Figure 5) may indicate an activation/deactivation at a low concentration of substrate. A similar activation pattern was found when using a dilution of cholesterol stock solution containing Triton X-100 and taurocholate in water or in water/Triton X-100 and nonionic surfactants and bile acid salts have been described to affect the kinetic behavior at particular enzyme to surfactant ratios [18]. The highest recorded specific activity for CgChoA was 15.5 U/mg.

### Enzymatic conversion of cholesterol to cholest-4-en-3-one

**Figure 6:**
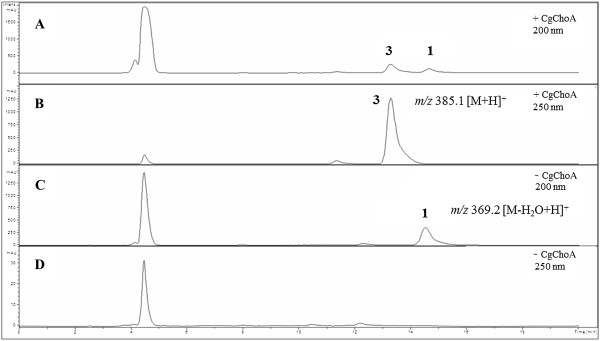
**Chromatograms after subjecting the extracted samples to HPLC-MS measurements: cholesterol (1) and oxidation product cholest-4-en-3-one (3).** Shown profiles are extracted at 200 and 250 nm from the DAD-signal for the reaction with CgChoA (**A** and **B**, respectively) and without enzyme (**C** and **D**, respectively); *m/z* signals are indicated.

Biocatalytic reactions were carried out using purified cholesterol oxidase and cholesterol at a concentration of 1 mM in the presence of 5% v/v Triton X-100. After 42 hours reaction time the product was extracted from the entire reaction batch with chloroform and analyzed. Figure 6 shows the traces monitored by HPLC-DAD at 200 and 250 nm for the enzymatic reaction. The product cholest-4-en-3-one (**3**), but not cholesterol (**1**) shows an absorbance at 250 nm. The peak of the chromatogram at 14.4 min at 200 nm corresponds to cholesterol with a mass signal of *m/z* 369.2 [M-H_2_O + H]^+^. The peak of the chromatogram at 13.1 min at 200 and 250 nm corresponds to cholest-4-en-3-one (**3**) with a mass signal of *m/z* 385.1 [M + H]^+^ and was only found in the reaction batch which contained cholesterol oxidase. Signals at 4.5 min derived from Triton X-100. There the mass pattern typical for PEG derivatives (series of mass signals differing in Δ*m/z* 44) was observed. The HPLC-MS analysis was performed for qualitative detection of the cholesterol conversion by CgChoA. Additional background signals could not be assigned to relevant compounds by MS. Commercially available cholesterol and cholest-4-en-3-one were used as reference substances.

## Discussion

Searching for novel cholesterol oxidases is of great interest in fields such as biosensing and enzymatic synthesis. The oxidation of cholesterol to cholest-4-en-3-one has been reported for cholesterol oxidase from whole cells of *C. gleum*, *Bacillus subtilis* and *Streptomyces sp*. [13,14,19]. Especially those enzymes with considerable low amino acid homology to already described ones may have novel optimal working conditions and thus be suitable for innovative applications.

With an approach similar to what reported for the production of other cholesterol oxidases (Table 1), the gene coding for CgChoA was cloned into pQE-30 and expressed in *E. coli* in the presence of pRARE2 to produce an enzyme with an N-terminal His-tag. The reduction of temperature to 16°C post induction was necessary to obtain soluble protein. The CgChoA was purified and found to occur presumably as a monomer, like cholesterol oxidase from *Brevibacterium* sp. and other bacteria [9,20]. A maximum specific cholesterol oxidase activity of 15.5 U/mg was found, which is in the same range of other recombinantly expressed cholesterol oxidases (Table 1). A maximum specific activity of 16.7 and 3.7 U/mg has been described for *Chromobacterium* sp. and *Brevibacterium* sp. (Table 1) respectively, both expressed without a His-tag [21,22]. Cholesterol oxidase from *Brevibacterium* sp. expressed with an N- or C-terminal His-tag, however, showed reduced activity for each construct when compared to the non-tagged enzyme [23]. It is therefore possible that a higher specific activity might be reached with a non His-tagged CgChoA and after more extensive purification. However, since the activity of the His-tagged enzyme was sufficient for characterization, we did not further investigate a non-tagged CgChoA.

**Table 1:** Comparison of different aspects concerning the production and selected properties of cholesterol oxidases

**Source (UniProt ID)**	**MW (kDa)**	**Expression system**^**a**^	**Purification steps**^**b**^	**Activity asssay**^**b**^	**Specific activity on cholesterol (U/mg)**	**Refs.**
*Brevibacterium sterolicum* (Q7SID9)	61.52	pET24b(+), *E. coli* BL21(DE3) pLysS, TB + 20 mL/L glycerol, 25/ 30°C	HiTrap (IMAC)	Potassium phosphate buffer pH 7.5 at 25°C (propan-2-ol and Thesit) monitoring H_2_O_2_ production using HRP and *o*-dianisidine	7 (crude extract)	[6,24]
*Brevibacterium sterolicum* nov. sp. ATCC21387	46.50	pUC19, *E. coli* MM294, LB, 30°C	Ammonium sulfate precipitation, DEAE-cellulose, Superose, hydroxyapatite	Sodium phosphate buffer pH 7.0, 37°C (Triton X-100), monitoring H_2_O_2_ production coupled to 4-aminoantipyrine and phenol *via* HRP	55.2	[25]
*Brevibacterium sterolicum* nov. sp. ATCC21387 (Q2I2N2)	59.07	pET28a(+), *E. coli* BL21(DE3), LB, 28°C	Affinity chromato-graphy, (riboflavin bound to, Sepharose 4B)	Quantifying H_2_O_2_ by coupling to HRP reaction with aminopyrine, 37°C	15.5	[20]
					(3.7, with His tag)	[22]
*Chromobacterium* sp. DS-1 (B5MGF)	63.00	pET-21d(+), *E. coli* Rosetta, LB, 30°C	Heat purification at 70°C for 30 min, DEAE-cellulose DE52	Sodium potassium phosphate buffer pH7.0, 30°C (sodium cholate, Triton X-100), 4-aminoantipyrine, phenol, HRP	16.7	[21,26]
*Chryseobacterium gleum* (D7VYA1)	59.00	pQE30 (pRARE2), *E. coli* JM109, modified M9, 16°C	HisTrap FF (IMAC), SEC on Superdex 200 pg	HPLC assay and coupled enzyme assay MOPS buffer, pH 6.75, 37°C, HRP and ABTS (Triton X-100 and taurocholic acid)	15.5	this study
*Streptomyces* sp. SA-COO (P12676)	58.99	pUC19, *E. coli* BL21(DE3)plysS, 2YT, 28°C	Butyl-Sepharose column chromatography, DEAE-cellulose column chromatography	Formation rate of H_2_O_2_ was monitored in a coupled assay with HRP and ABTS, 37°C (Triton X-100, BSA)	-	[10,27,28]

Data taken from the Brenda database ( http://www.brenda-enzymes.org/), RCSB Protein data bank (pdb) and indicated references (refs.).

^a^vector, host, expression strain, cultivation medium, cultivation conditions.

^b^*Abbreviations:ABTS* 2,2'-azino-bis(3-ethylbenzothiazoline-6-sulphonic acid), *BSA* bovine serum albumin, *HRP* horseradish peroxidase, *IMAC* Immobilized metal ion affinity chromatography, *SEC* size-exclusion chromatography.

The recombinant CgChoA was active between pH 4–8 with optimal activity in the neutral range similarly to other cholesterol oxidases (Table 1), e.g. at pH 6.75 using 0.011 M MOPS buffer for the coupled HRP assay. At higher concentrations of MOPS, the activity declined steadily at any of the 6 pH values measured. MOPS buffer with a pH lower than 6.75 has not been tested as it buffers only between 6.5 and 8. A temperature optimum between 32°C and 40°C was found, which is in the range of the cholesterol oxidase from *Corynebacterium cholesterolicum*, but lower than that of *Streptomyces violascens* or *Brevibacterium* sp. enzymes, which showed optimum activity at around 50°C [5]. The activity data obtained when the substrate was dissolved in the presence of Triton X-100 and/or water only could not be fitted to the Michaelis-Menten equation, which is only applicable for enzymatic reactions in homogeneous solutions and therefore cannot be directly adapted to the heterogeneous reaction conditions that were applied here*.* The presence of the anionic surfactant taurocholate proved to affect the measured activity and an apparent *K*_m_ of 0.5 mM was obtained for CgChoA and the substrate cholesterol. We cannot exclude for taurocholate an effect not only regarding an improved substrate solubilisation, and thus enhanced accessibility to the enzyme, but also an effect on the enzyme itself. In summary, the anionic surfactant taurocholate is sufficient as additive for monitoring the enzyme activity of CgChoA with regard to the natural substrate cholesterol, while the presence of the non-ionic additive Triton X-100 did not affect the general kinetic behaviour. These data may be of special interest for developing biosensors for samples with at low cholesterol content as dilution in the presence of taurocholate might provide a linear correlation between the substrate concentration and the signal measured.

## Conclusions

The cholesterol oxidase CgChoA from *C. gleum* was successfully expressed in *E. coli* JM109 co-transformed with pCgChoA and pRARE2. The CgChoA carrying an N-terminal His-tag was purified and subjected to a pH and temperature screen. The highest specific activity was determined to be 15.5 U/mg. Michaelis-Menten type kinetics could only be observed in the presence of taurocholate as single surfactant within the enzymatic assay. The CgChoA cholesterol oxidation product was identified as cholest-4-en-3-one by direct and rapid detection *via* HPLC-MS. The rapid and robust HPLC-MS assay developed in this study enables a more detailed study of CgChoA and other cholesterol oxidases. The described enzyme complements the set of available cholesterol oxidases for diverse applications such as bionsensing and synthesis of intermediates for drug synthesis. As successful biotransformation employing *C. gleum* as host organism has already been demonstrated [14], the future engineering of CgChoA for a broader substrate specificity might enable the application of this enzyme for the conversion of other steroid compounds.

## Methods

### Bacterial strains

*Chryseobacterium gleum* DSM 16776 was obtained from the German collection of microorganisms (DSMZ). *E. coli* strain JM109 [genotype *endA*1 *recA*1, *gyrA*96, *thi*, *hsdR*17,(r_K_^-^, m_K_^+^), *relA*1, *supE*44, λ^-^, Δ*(lac-proAB)*, (F', *traD*36, *proAB*, *lacI*^q^ZΔM15)] and the pQE-30 expression vector were purchased from Promega (Madison, USA) and Qiagen (Valencia, USA), respectively. The origin of replication in pQE-30 is ColE1 (pBR322) and transcription of the inserted gene is controlled by the bacteriophage T5 promoter (recognized by the *E. coli* housekeeping RNA polymerase) and two *lac* operator sequences (conferring inducibility by IPTG). For efficient repression the host strain JM109 which over-expresses the LacI repressor was used. JM109 was transformed with the plasmid pRARE2, which contains the tRNA genes *argU*, *argW, ileX, glyT, leuW, proL, metT, thrT, tyrU, thrU* and *argX.* The usage of the rare codons AGG, AGA, AUA, CUA, CCC, GGA and CGG is thereby supplemented. The plasmid was isolated from Rosetta2 (DE3) (Merck Chemicals, UK) (F^-^*ompT hsdS*_B_(r_B_^-^ m_B_^-^) *gal dcm* (DE3) pRARE2) cells. The resulting chloramphenicol-resistant strain JM109-pRARE2 was the expression host.

### Cloning of *choA* from *C. gleum*

The putative cholesterol oxidase gene *choA* of *C. gleum* was identified by Protein blast (NCBI website) using the cholesterol oxidase sequence of *Streptomyces* sp. (UniProt accession no. P12676) as search template. The cholesterol oxidase gene of *C. gleum* (accession no. ACKQ02000004) was PCR amplified from genomic DNA with forward primer 5’ GCG *GCA TGC* GAC AGA AAA AAA TTC ATC AGG ACA AGT GC 3’ (introducing a *Sph*I site around the native start codon) and reverse primer 5’ CCG *AAG CTT* TTA ACC CAG GTT AAA TTC ATT TTG CCG G 3’ (introducing a *Hin*dIII site after the native stop codon). PCR was performed with high fidelity Phusion polymerase (New England Biolabs, Ipswich, USA) and a diluted solution of genomic DNA of *C. gleum* DSM 16776 as template source. Genomic DNA was isolated using the GenElute Bacterial genomic DNA kit (Sigma-Aldrich, CH). Plasmid DNA and PCR products were purified using the Gene Jet Plasmid Miniprep Kit (Fermentas) and the GenElute PCR clean-up kit (Sigma-Aldrich). DNA from agarose gels was recovered using the GenElute Gel extraction kit (Sigma-Aldrich, CH). The 1596 bp PCR product was cloned into the pQE-30 expression vector in frame with a sequence coding for an N-terminal hexa-histidine tag to allow purification by immobilized metal affinity chromatography. The in frame cloning of the *choA* gene from *C. gleum* DSM 16776 in the final expression plasmid pCgChoA was confirmed by DNA sequencing (GATC, Germany).

### Cell cultivation and protein purification

*C. gleum* DSM 16776 was grown overnight at 30°C at 180 rpm in trypticase soy yeast extract medium (trypticase soy broth 30 g/L, yeast extract 3 g/L). *E. coli* JM109-pRARE2 was transformed with pCgChoA. Expression of the recombinant protein was performed in medium containing 1× M9 salts, 20 g/L N-Z-amine, 20 g/L glycerol, 1 mM MgSO_4,_ 1 mM MgCl_2_, 100 μM CaCl_2_, 100 μM thiamine, 0.025% glucose and trace metal mixture [29]. A 100 mL overnight culture was grown from a single colony (LB agar) and used to inoculate 700 mL of medium (dilution 1:50). The culture was grown at 37°C with shaking at 180 rpm. At an OD_600_ = 0.8, protein production was induced at 0.1 mM isopropyl thio-β-D-galactoside (IPTG). At the same time, the temperature and shaking were reduced to 16°C and 120 rpm for 16–18 hours. For plasmid selection 100 μg/mL ampicillin and 20 μg/mL chloramphenicol were added to plates and liquid media. For protein purification cells were harvested by centrifugation at 4°C for 30 min at 4,495 × g, washed in 0.1 M sodium phosphate buffer pH 7, centrifuged again and subsequently stored at -20°C. Frozen cells were thawed on ice and resuspended in 0.1 M sodium phosphate buffer pH 7 with 20 mM imidazole and 0.5 M sodium chloride (buffer A) containing 1 mg/mL lysozyme and protease inhibitor mix (Roche Complete Protease Inhibitor Mix, EDTA-free) and re-frozen at -80°C. Cells were thawed, Benzonase Nuclease (Roche) was added and the suspension incubated for 1 h at 37°C at 120 rpm. The suspension was subjected to twelve 10 s rounds of sonication with a Branson sonicator equipped with a microtip at a setting of 80%. Cellular debris was removed by centrifugation at 4°C for 40 min, 47,000 × g. Purification was performed on an Äkta purifier FPLC system (GE-Healthcare). The sample was loaded onto a 1 mL HisTrap FF chromatography column (GE-Healthcare), previously equilibrated with buffer A. Proteins were eluted with a imidazole gradient from 0 to 1 M. Fractions displaying cholesterol activity were pooled and concentrated by ultrafiltration using a 30 kDa cut-off. The sample was loaded onto a Superdex 200 column (GE-Healthcare), previously equilibrated with 20 mM MOPS buffer pH 6.75 containing 0.1 M NaCl. Fractions with cholesterol oxidase activity were pooled and concentrated by ultrafiltration. The purity of the sample was analyzed by SDS-PAGE using a 10% polyacrylamide gel. The gel filtration kit (GE-Healthcare) was used to calibrate a Superdex 200 column with high and low molecular weight standards, previously equilibrated with 20 mM MOPS buffer (pH 6.75) containing 0.1 M NaCl.

### Activity assay and protein determination

A 27.2 mM stock solution/dispersion of cholesterol was prepared and diluted in water in the presence or absence of 5% (v/v) Triton X-100, 2.9% (w/v) of taurocholic acid sodium salt (Sigma Aldrich), and a combinations thereof. Cholesterol oxidase activity was assayed by quantifying H_2_O_2_ formation from the coupling reaction with HRP. The activity assay mixture contained 40 μL of cholesterol at the selected concentration, 10 μL of HRP (concentration 1 mg/mL, in ddH_2_O), 10 μL of ABTS (concentration 10 mM, in ddH_2_O), 110 μL of 0.011 M MOPS buffer pre-heated to 37°C, and 30 μL of the purified enzyme preparation in a total volume of 200 μL. The spectrophotometric cholesterol activity assay was carried out in a 96-well plate using a BioTek Synergy Mx spectrophotometer. ABTS (0.6 mM), pyrogallol red (0.15 mM) and *o*-dianisidine (0.5 mM) were used as substrates for the HRP coupled assay using 0.011 M MOPS buffer pH 6.75 at 37°C. The reaction was started by adding cholesterol oxidase and followed for oxidation of ABTS at 420 nm (ε = 36 000 M^-1^ cm^-1^), of pyrogallol red at 550 nm (ε = 30 900 M^-1^ cm^-1^) and of *o*-dianisidine at 440 nm (ε = 13 000 M^-1^ cm^-1^). Kinetic parameters of cholesterol oxidase samples were determined between 0.17 μM – 5.5 mM cholesterol at 35°C, and results were analyzed with the Enzyme Kinetics Module of the software SigmaPlot (Systat Software Inc., CA, USA).

Cholesterol activity as a function of the pH was recorded *via* the HRP coupled assay with 0.5 mM ABTS and 0.55 mM cholesterol using Teorell-Stenhagen buffer (pH 4.0, 5.0, 6.0, 7.0, 7.5, 8.0, and 8.5), 0.1 M sodium phosphate buffer (pH 6.0, 6.4, 7.0, 7.5, and 7.8), 0.11 M MOPS pH 6.75, 0.1 M potassium phosphate buffer (pH 6.0, 6.5, 7.0, 7.5, and 7.8), and McIlvaine buffer (pH 4.0, 5.0, 6.0, 7.0, 7.5, and 8.0). Further 0.55 M, 0.275 M, 0.11 M 0.055 M, 0.0275 M and 0.011 M MOPS buffers (pH 6.75, 7, 7.25, 7.5, 7.75, and 8.0) were tested. The temperature optimum was recorded between 24 and 48°C following ABTS oxidation with 0.55 mM cholesterol, in an assay volume of 3 mL using a magnetically stirred, temperature-controlled cuvette device using a Varian Cary 50 Bio spectrophotometer. Total protein concentration was determined by the method of Bradford, with bovine serum albumin as standard.

### Biocatalytic reactions

The substrate cholesterol was added from a stock solution, which was made up as described above (containing Triton X-100 and taurocholate), to a final concentration of 1 mM in 0.011 M MOPS buffer pH 6.75. The reaction was adjusted to 600 μL and 0.04 mg of purified cholesterol oxidase from *C. gleum* was added (0.67 U/mL). For the blank reaction water was used instead of enzyme solution. All reactions were prepared in triplicate. The reaction mixture was left shaking at 250 rpm at 30°C for 42 hours in 3 mL screw cap glass vials.

### Analysis of cholesterol and cholest-4-en-3-one by HPLC-MS

The total reaction was extracted on 1 mL chloroform. After evaporation of the solvent at room temperature, the product was dissolved in the solvent, which was the same as the mobile phase used for HPLC. 10 μL of the analyte sample were injected into a Phenomenex Gemini® 5 μ C18 110 Å column (250 × 4.6 mm, 5 micron), and chromatography under isocratic conditions was performed using methanol:water 100:2 (v/v) at a flow rate of 0.8 mL/min at room temperature. Cholesterol and cholest-4-en-3-one were purchased from Sigma-Aldrich and used as reference. Product formation was monitored at 200 and 250 nm, whereas cholesterol was detected at 200 nm. The Agilent HPLC 1100 system equipped with a DAD was coupled to an esquireHCT ion trap mass spectrometer (Bruker. Germany), and an atmospheric pressure chemical ionization (APCI) source was operated in the positive ion mode. Conditions were as follows: scan range, *m/z* 50–600; dry gas flow of 11 L/min, nebulizer pressure 35 psi, drying gas temperature 320°C and the APCI heater temperature was 350°C. The extracted ion current (EIC) signals were deduced based on the exact masses for protonated cholesterol after water elimination (*m/z* 369.34) as well as for the protonated oxidation product cholest-4-en-3-one (*m/z* 385.34).

## Abbreviations

ABTS: 2,2'-Azino-bis 3-ethylbenzothiazoline-6-sulphonic acid; APCI: Atmospheric pressure chemical ionization; CgChoA: Cholesterol oxidase from *Chryseobacterium gleum*; HRP: Horseradish peroxidase.

## Competing interests

The authors declare that they have no competing interests.

## Authors’ contributions

RR participated in the design of the study, carried out the experiment and in writing the manuscript. GF confirmed the kinetic study and contributed to the preparation of the revised manuscript. MR participated in the design of the study, carried out the HPLC-MS analysis and helped to draft the manuscript. LTM provided financial and administrative support and participated in the design of the study and writing the manuscript. All authors read and approved the final manuscript.
